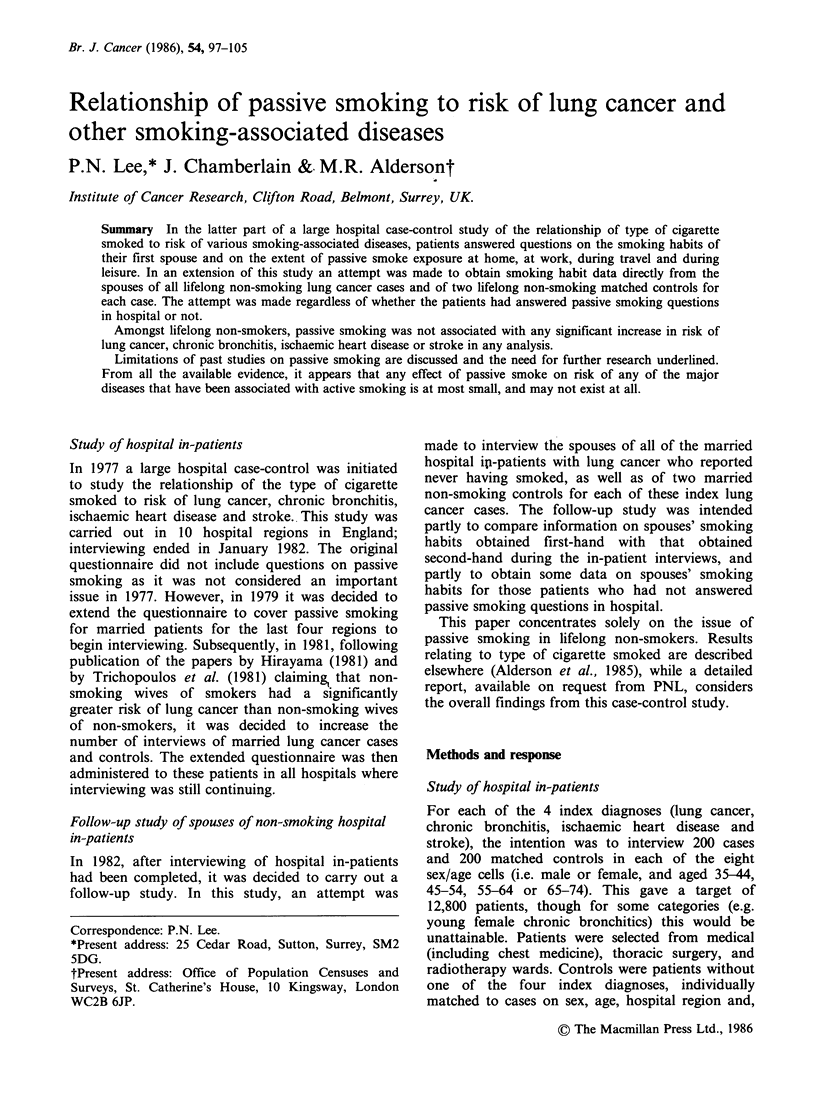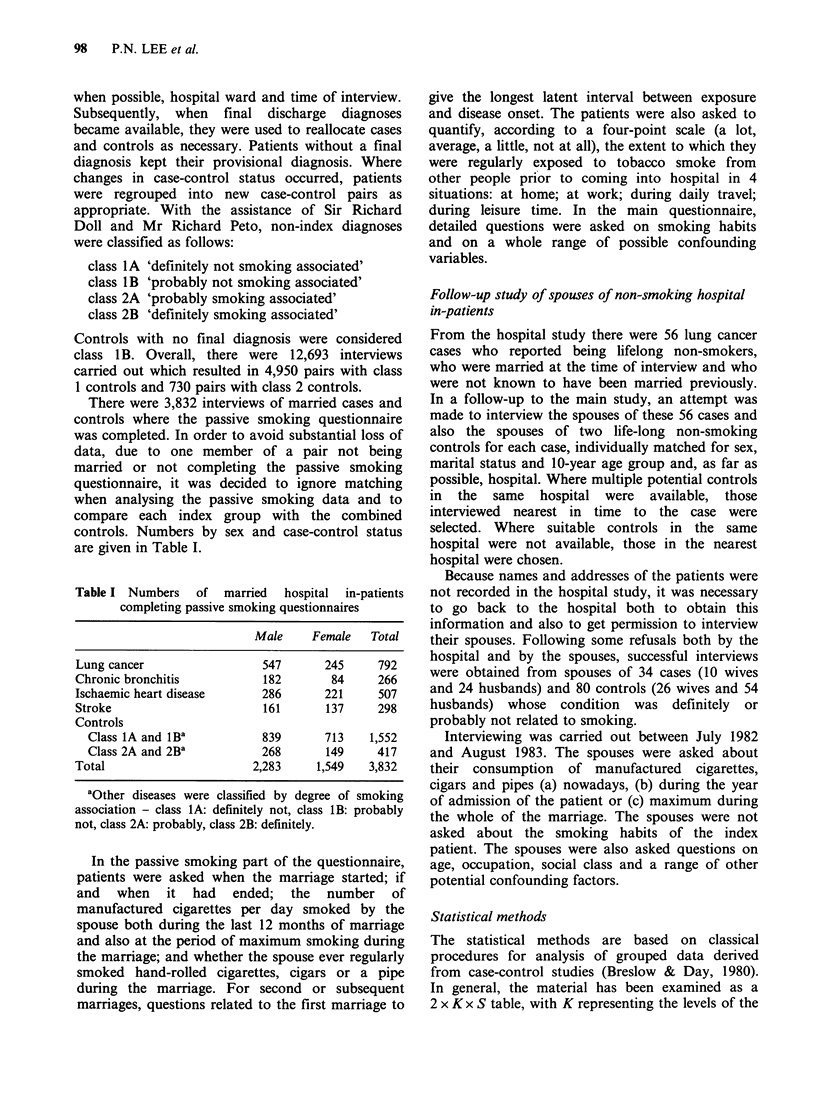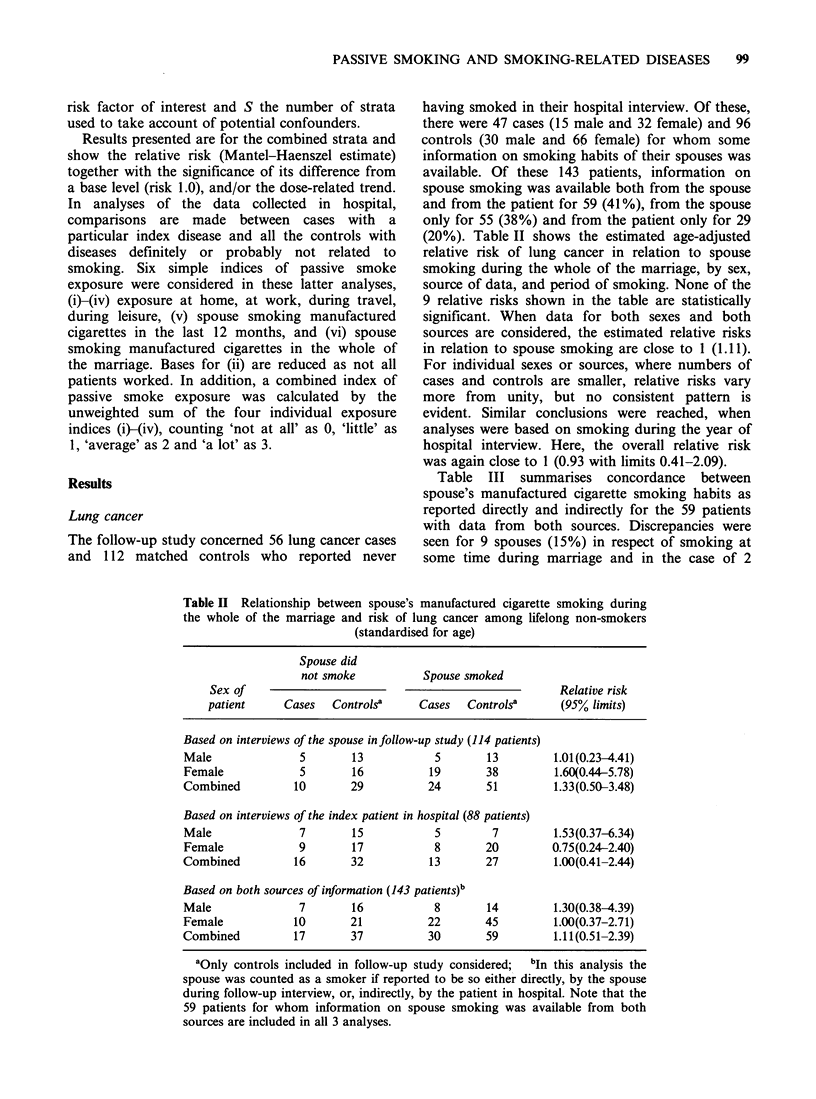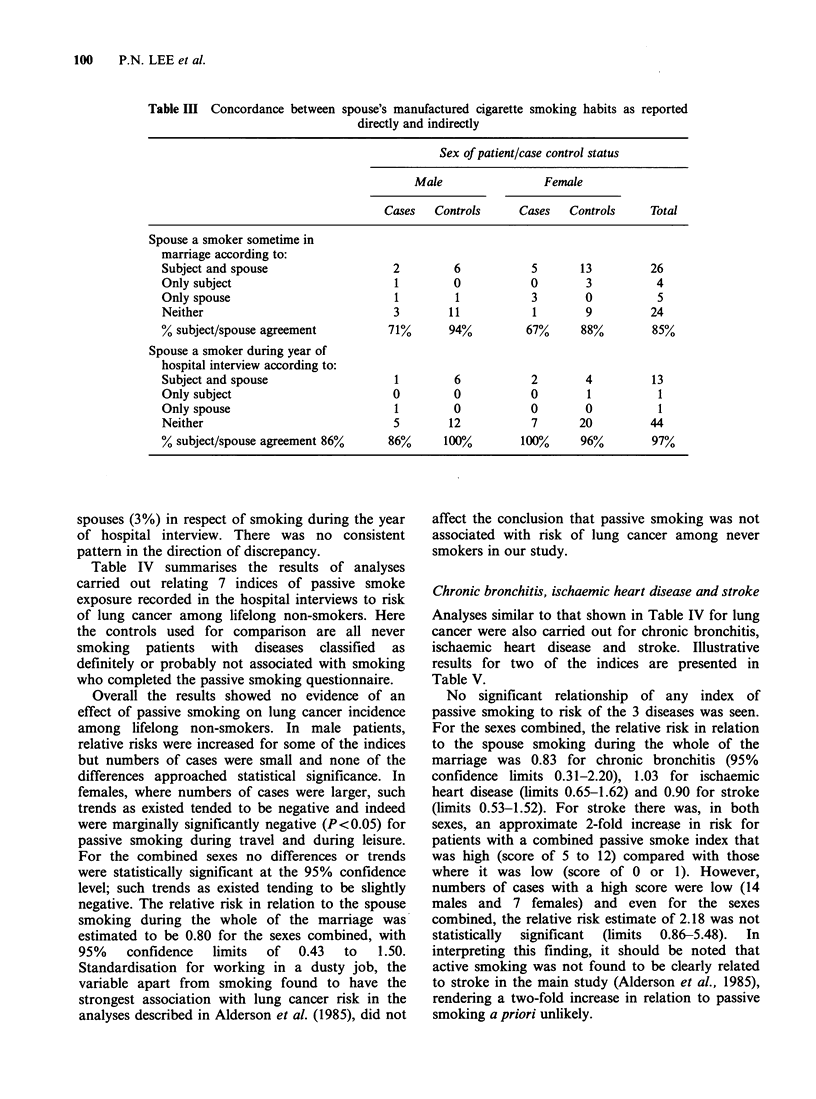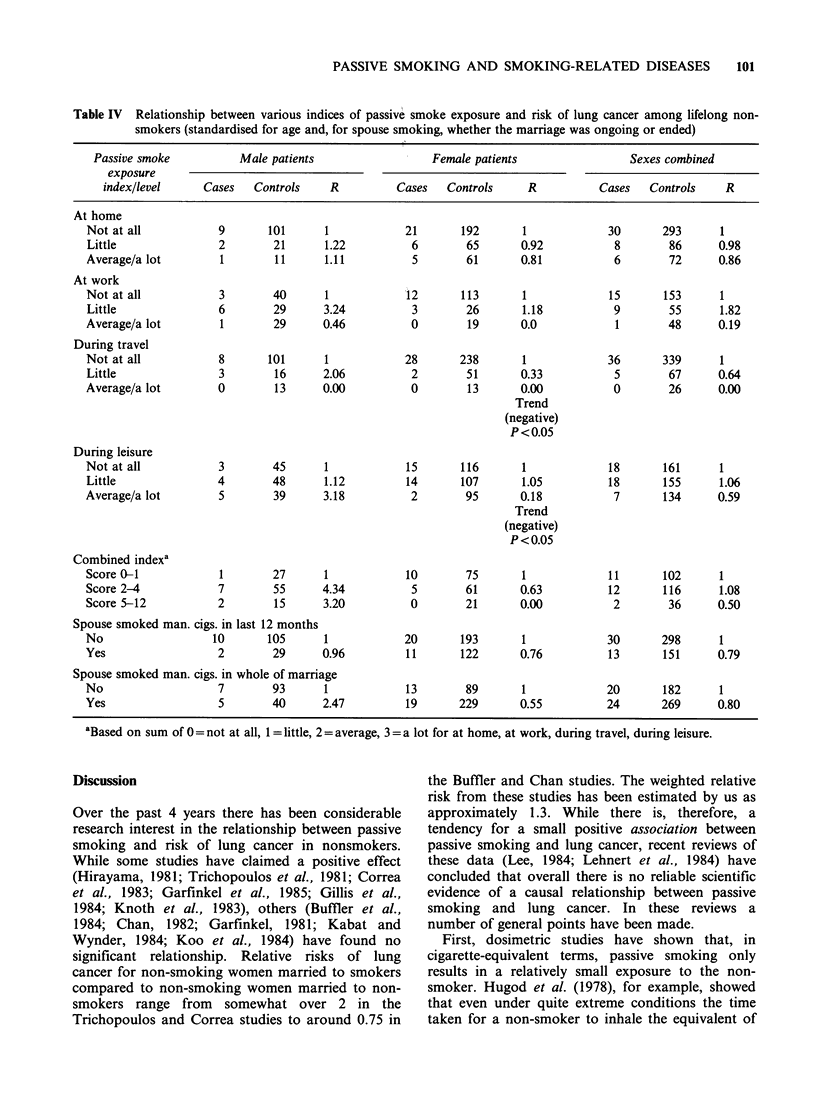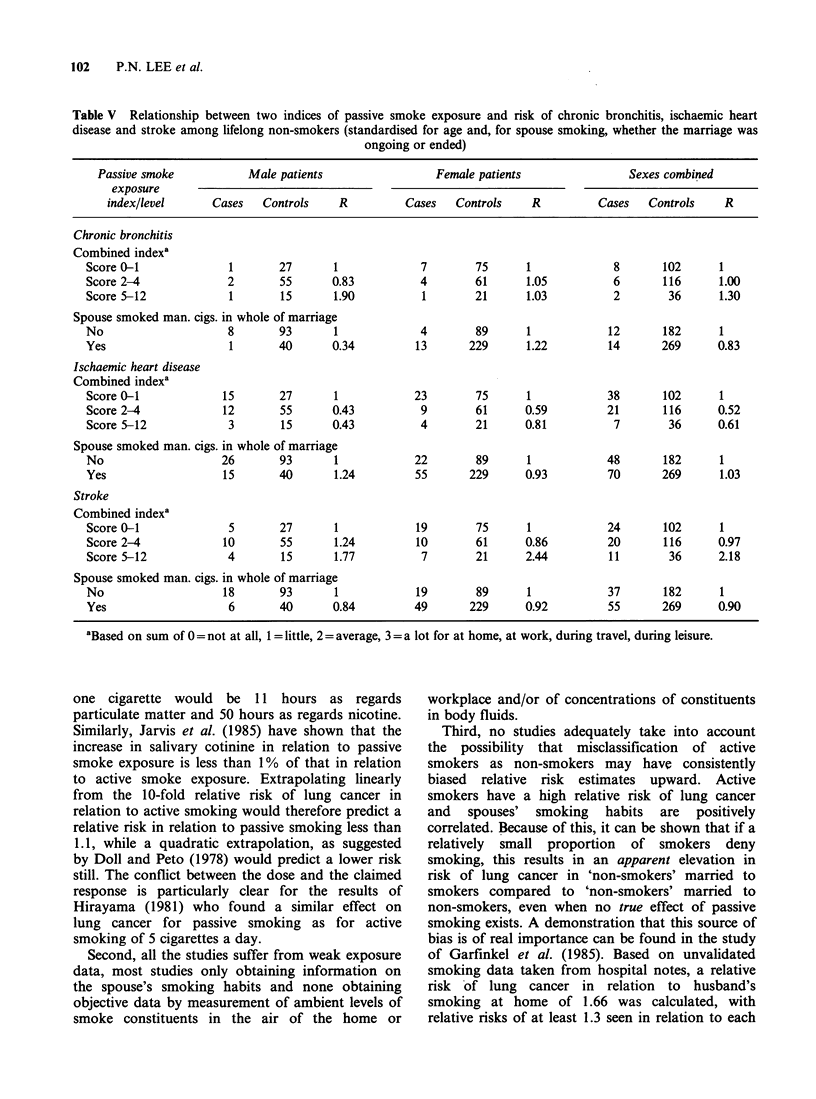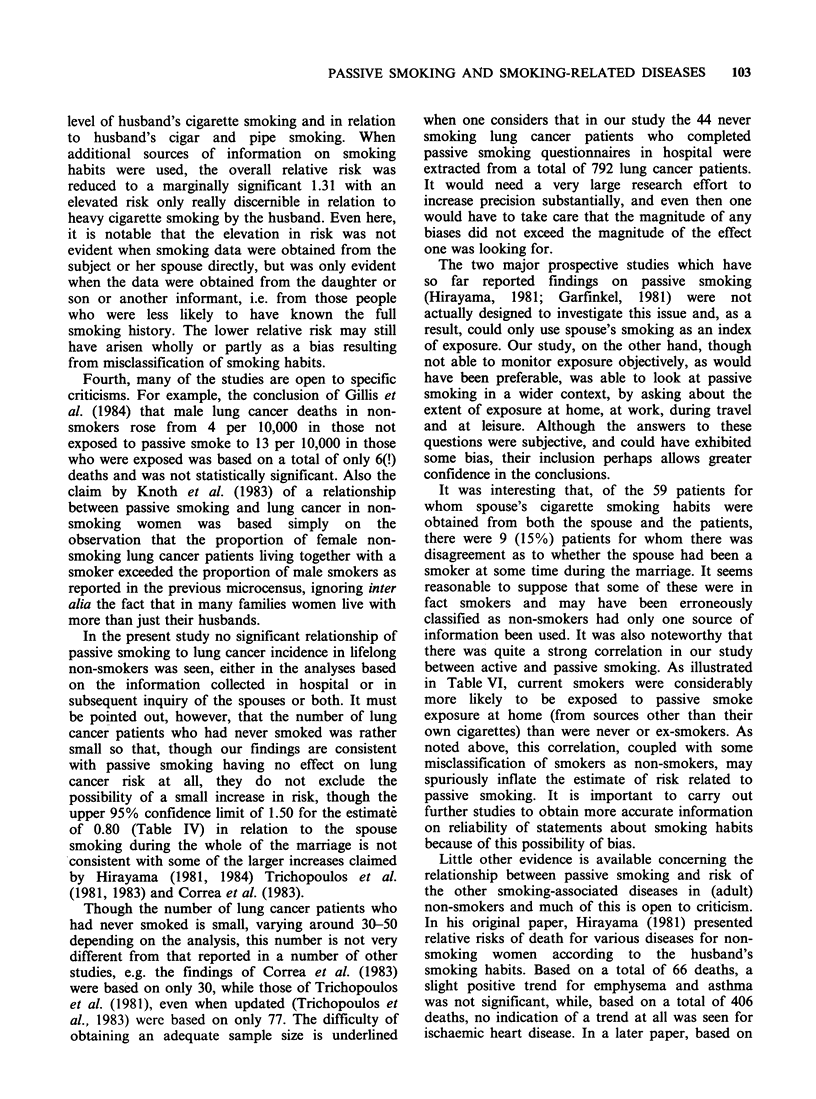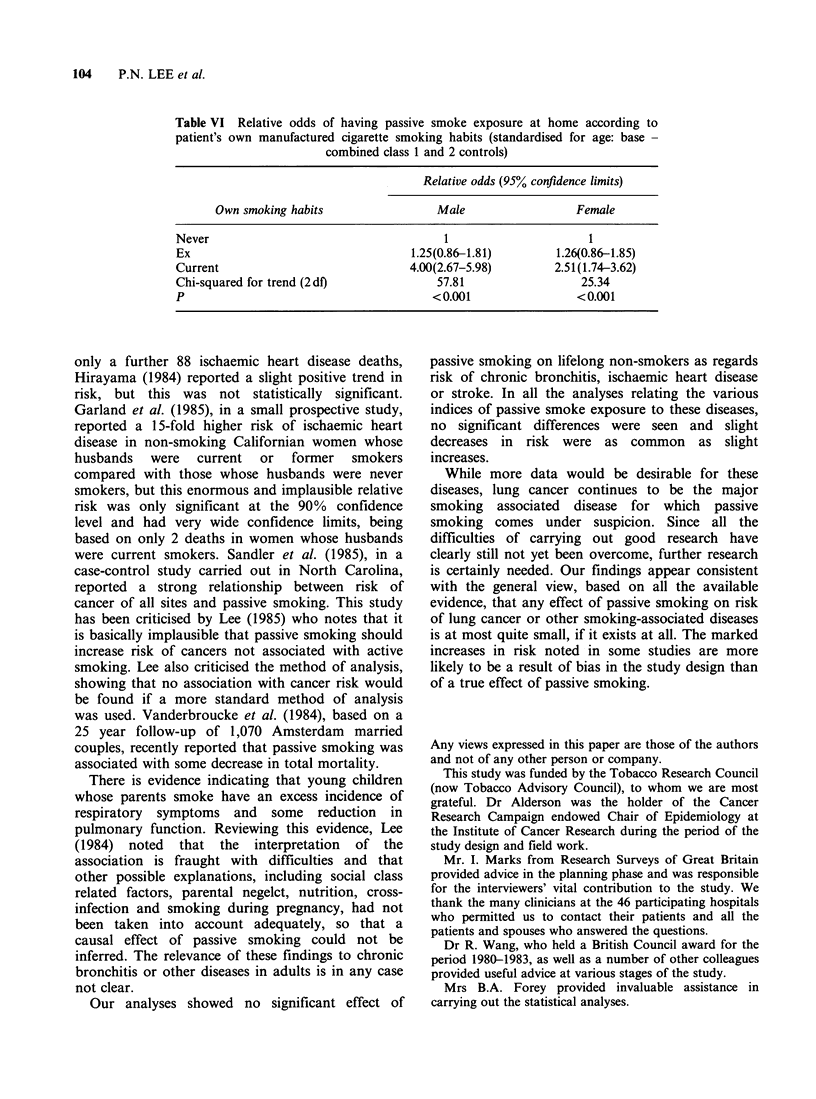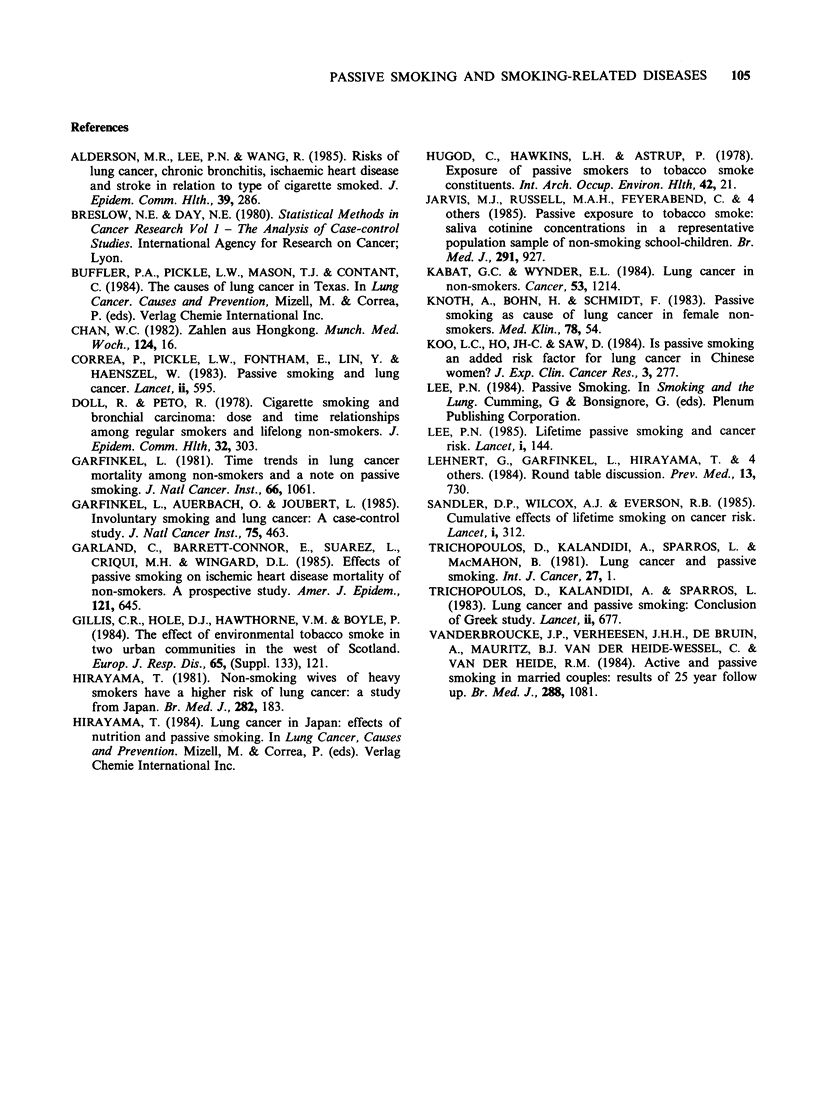# Relationship of passive smoking to risk of lung cancer and other smoking-associated diseases.

**DOI:** 10.1038/bjc.1986.157

**Published:** 1986-07

**Authors:** P. N. Lee, J. Chamberlain, M. R. Alderson

## Abstract

In the latter part of a large hospital case-control study of the relationship of type of cigarette smoked to risk of various smoking-associated diseases, patients answered questions on the smoking habits of their first spouse and on the extent of passive smoke exposure at home, at work, during travel and during leisure. In an extension of this study an attempt was made to obtain smoking habit data directly from the spouses of all lifelong non-smoking lung cancer cases and of two lifelong non-smoking matched controls for each case. The attempt was made regardless of whether the patients had answered passive smoking questions in hospital or not. Amongst lifelong non-smokers, passive smoking was not associated with any significant increase in risk of lung cancer, chronic bronchitis, ischaemic heart disease or stroke in any analysis. Limitations of past studies on passive smoking are discussed and the need for further research underlined. From all the available evidence, it appears that any effect of passive smoke on risk of any of the major diseases that have been associated with active smoking is at most small, and may not exist at all.


*Br. J. Cancer* (1986), **54**, 97–105

### Relationship of passive smoking to risk of lung cancer and other smoking-associated diseases

P.N. Lee,* J. Chamberlain & M.R. Alderson†

*Institute of Cancer Research, Clifton Road, Belmont, Surrey, UK.*

**Summary** In the latter part of a large hospital case-control study of the relationship of type of cigarette smoked to risk of various smoking-associated diseases, patients answered questions on the smoking habits of their first spouse and on the extent of passive smoke exposure at home, at work, during travel and during leisure. In an extension of this study an attempt was made to obtain smoking habit data directly from the spouses of all lifelong non-smoking lung cancer cases and of two lifelong non-smoking matched controls for each case. The attempt was made regardless of whether the patients had answered passive smoking questions in hospital or not.

Amongst lifelong non-smokers, passive smoking was not associated with any significant increase in risk of lung cancer, chronic bronchitis, ischaemic heart disease or stroke in any analysis.

Limitations of past studies on passive smoking are discussed and the need for further research underlined. From all the available evidence, it appears that any effect of passive smoke on risk of any of the major diseases that have been associated with active smoking is at most small, and may not exist at all.

*Study of hospital in-patients*

In 1977 a large hospital case-control was initiated to study the relationship of the type of cigarette smoked to risk of lung cancer, chronic bronchitis, ischaemic heart disease and stroke. This study was carried out in 10 hospital regions in England; interviewing ended in January 1982. The original questionnaire did not include questions on passive smoking as it was not considered an important issue in 1977. However, in 1979 it was decided to extend the questionnaire to cover passive smoking for married patients for the last four regions to begin interviewing. Subsequently, in 1981, following publication of the papers by Hirayama (1981) and by Trichopoulos *et al.* (1981) claiming that non-smoking wives of smokers had a significantly greater risk of lung cancer than non-smoking wives of non-smokers, it was decided to increase the number of interviews of married lung cancer cases and controls. The extended questionnaire was then administered to these patients in all hospitals where interviewing was still continuing.

*Follow-up study of spouses of non-smoking hospital in-patients*

In 1982, after interviewing of hospital in-patients had been completed, it was decided to carry out a follow-up study. In this study, an attempt was made to interview the spouses of all of the married hospital in-patients with lung cancer who reported never having smoked, as well as of two married non-smoking controls for each of these index lung cancer cases. The follow-up study was intended partly to compare information on spouses' smoking habits obtained first-hand with that obtained second-hand during the in-patient interviews, and partly to obtain some data on spouses' smoking habits for those patients who had not answered passive smoking questions in hospital.

This paper concentrates solely on the issue of passive smoking in lifelong non-smokers. Results relating to type of cigarette smoked are described elsewhere (Alderson *et al.*, 1985), while a detailed report, available on request from PNL, considers the overall findings from this case-control study.

### Methods and response

*Study of hospital in-patients*

For each of the 4 index diagnoses (lung cancer, chronic bronchitis, ischaemic heart disease and stroke), the intention was to interview 200 cases and 200 matched controls in each of the eight sex/age cells (i.e. male or female, and aged 35–44, 45–54, 55–64 or 65–74). This gave a target of 12,800 patients, though for some categories (e.g. young female chronic bronchitics) this would be unattainable. Patients were selected from medical (including chest medicine), thoracic surgery, and radiotherapy wards. Controls were patients without one of the four index diagnoses, individually matched to cases on sex, age, hospital region and,

Correspondence: P.N. Lee.
*Present address: 25 Cedar Road, Sutton, Surrey, SM2 5DG.
†Present address: Office of Population Censuses and Surveys, St. Catherine's House, 10 Kingsway, London WC2B 6JP.

© The Macmillan Press Ltd., 1986

when possible, hospital ward and time of interview. Subsequently, when final discharge diagnoses became available, they were used to reallocate cases and controls as necessary. Patients without a final diagnosis kept their provisional diagnosis. Where changes in case-control status occurred, patients were regrouped into new case-control pairs as appropriate. With the assistance of Sir Richard Doll and Mr Richard Peto, non-index diagnoses were classified as follows:

class 1A 'definitely not smoking associated'
class 1B 'probably not smoking associated'
class 2A 'probably smoking associated'
class 2B 'definitely smoking associated'

Controls with no final diagnosis were considered class 1B. Overall, there were 12,693 interviews carried out which resulted in 4,950 pairs with class 1 controls and 730 pairs with class 2 controls.

There were 3,832 interviews of married cases and controls where the passive smoking questionnaire was completed. In order to avoid substantial loss of data, due to one member of a pair not being married or not completing the passive smoking questionnaire, it was decided to ignore matching when analysing the passive smoking data and to compare each index group with the combined controls. Numbers by sex and case-control status are given in Table I.

**Table I** Numbers of married hospital in-patients completing passive smoking questionnaires

| | *Male* | *Female* | *Total* |
|---|---|---|---|
| Lung cancer | 547 | 245 | 792 |
| Chronic bronchitis | 182 | 84 | 266 |
| Ischaemic heart disease | 286 | 221 | 507 |
| Stroke | 161 | 137 | 298 |
| Controls | | | |
| Class 1A and 1B[a] | 839 | 713 | 1,552 |
| Class 2A and 2B[a] | 268 | 149 | 417 |
| Total | 2,283 | 1,549 | 3,832 |

[a]Other diseases were classified by degree of smoking association – class 1A: definitely not, class 1B: probably not, class 2A: probably, class 2B: definitely.

In the passive smoking part of the questionnaire, patients were asked when the marriage started; if and when it had ended; the number of manufactured cigarettes per day smoked by the spouse both during the last 12 months of marriage and also at the period of maximum smoking during the marriage; and whether the spouse ever regularly smoked hand-rolled cigarettes, cigars or a pipe during the marriage. For second or subsequent marriages, questions related to the first marriage to give the longest latent interval between exposure and disease onset. The patients were also asked to quantify, according to a four-point scale (a lot, average, a little, not at all), the extent to which they were regularly exposed to tobacco smoke from other people prior to coming into hospital in 4 situations: at home; at work; during daily travel; during leisure time. In the main questionnaire, detailed questions were asked on smoking habits and on a whole range of possible confounding variables.

### *Follow-up study of spouses of non-smoking hospital in-patients*

From the hospital study there were 56 lung cancer cases who reported being lifelong non-smokers, who were married at the time of interview and who were not known to have been married previously. In a follow-up to the main study, an attempt was made to interview the spouses of these 56 cases and also the spouses of two life-long non-smoking controls for each case, individually matched for sex, marital status and 10-year age group and, as far as possible, hospital. Where multiple potential controls in the same hospital were available, those interviewed nearest in time to the case were selected. Where suitable controls in the same hospital were not available, those in the nearest hospital were chosen.

Because names and addresses of the patients were not recorded in the hospital study, it was necessary to go back to the hospital both to obtain this information and also to get permission to interview their spouses. Following some refusals both by the hospital and by the spouses, successful interviews were obtained from spouses of 34 cases (10 wives and 24 husbands) and 80 controls (26 wives and 54 husbands) whose condition was definitely or probably not related to smoking.

Interviewing was carried out between July 1982 and August 1983. The spouses were asked about their consumption of manufactured cigarettes, cigars and pipes (a) nowadays, (b) during the year of admission of the patient or (c) maximum during the whole of the marriage. The spouses were not asked about the smoking habits of the index patient. The spouses were also asked questions on age, occupation, social class and a range of other potential confounding factors.

### *Statistical methods*

The statistical methods are based on classical procedures for analysis of grouped data derived from case-control studies (Breslow & Day, 1980). In general, the material has been examined as a $2 \times K \times S$ table, with $K$ representing the levels of the risk factor of interest and $S$ the number of strata used to take account of potential confounders.

Results presented are for the combined strata and show the relative risk (Mantel–Haenszel estimate) together with the significance of its difference from a base level (risk 1.0), and/or the dose-related trend. In analyses of the data collected in hospital, comparisons are made between cases with a particular index disease and all the controls with diseases definitely or probably not related to smoking. Six simple indices of passive smoke exposure were considered in these latter analyses, (i)–(iv) exposure at home, at work, during travel, during leisure, (v) spouse smoking manufactured cigarettes in the last 12 months, and (vi) spouse smoking manufactured cigarettes in the whole of the marriage. Bases for (ii) are reduced as not all patients worked. In addition, a combined index of passive smoke exposure was calculated by the unweighted sum of the four individual exposure indices (i)–(iv), counting 'not at all' as 0, 'little' as 1, 'average' as 2 and 'a lot' as 3.

### Results

### *Lung cancer*

The follow-up study concerned 56 lung cancer cases and 112 matched controls who reported never having smoked in their hospital interview. Of these, there were 47 cases (15 male and 32 female) and 96 controls (30 male and 66 female) for whom some information on smoking habits of their spouses was available. Of these 143 patients, information on spouse smoking was available both from the spouse and from the patient for 59 (41%), from the spouse only for 55 (38%) and from the patient only for 29 (20%). Table II shows the estimated age-adjusted relative risk of lung cancer in relation to spouse smoking during the whole of the marriage, by sex, source of data, and period of smoking. None of the 9 relative risks shown in the table are statistically significant. When data for both sexes and both sources are considered, the estimated relative risks in relation to spouse smoking are close to 1 (1.11). For individual sexes or sources, where numbers of cases and controls are smaller, relative risks vary more from unity, but no consistent pattern is evident. Similar conclusions were reached, when analyses were based on smoking during the year of hospital interview. Here, the overall relative risk was again close to 1 (0.93 with limits 0.41–2.09).

Table III summarises concordance between spouse's manufactured cigarette smoking habits as reported directly and indirectly for the 59 patients with data from both sources. Discrepancies were seen for 9 spouses (15%) in respect of smoking at some time during marriage and in the case of 2

**Table II** Relationship between spouse's manufactured cigarette smoking during the whole of the marriage and risk of lung cancer among lifelong non-smokers (standardised for age)

| *Sex of patient* | *Spouse did not smoke* | | *Spouse smoked* | | *Relative risk (95% limits)* |
|---|---|---|---|---|---|
| | *Cases* | *Controls*[a] | *Cases* | *Controls*[a] | |
| *Based on interviews of the spouse in follow-up study (114 patients)* | | | | | |
| Male | 5 | 13 | 5 | 13 | 1.01(0.23–4.41) |
| Female | 5 | 16 | 19 | 38 | 1.60(0.44–5.78) |
| Combined | 10 | 29 | 24 | 51 | 1.33(0.50–3.48) |
| *Based on interviews of the index patient in hospital (88 patients)* | | | | | |
| Male | 7 | 15 | 5 | 7 | 1.53(0.37–6.34) |
| Female | 9 | 17 | 8 | 20 | 0.75(0.24–2.40) |
| Combined | 16 | 32 | 13 | 27 | 1.00(0.41–2.44) |
| *Based on both sources of information (143 patients)*[b] | | | | | |
| Male | 7 | 16 | 8 | 14 | 1.30(0.38–4.39) |
| Female | 10 | 21 | 22 | 45 | 1.00(0.37–2.71) |
| Combined | 17 | 37 | 30 | 59 | 1.11(0.51–2.39) |

[a]Only controls included in follow-up study considered; [b]In this analysis the spouse was counted as a smoker if reported to be so either directly, by the spouse during follow-up interview, or, indirectly, by the patient in hospital. Note that the 59 patients for whom information on spouse smoking was available from both sources are included in all 3 analyses.

**Table III** Concordance between spouse's manufactured cigarette smoking habits as reported directly and indirectly

| | *Sex of patient/case control status* | | | | |
|---|---|---|---|---|---|
| | *Male* | | *Female* | | |
| | *Cases* | *Controls* | *Cases* | *Controls* | *Total* |
| Spouse a smoker sometime in marriage according to: | | | | | |
| Subject and spouse | 2 | 6 | 5 | 13 | 26 |
| Only subject | 1 | 0 | 0 | 3 | 4 |
| Only spouse | 1 | 1 | 3 | 0 | 5 |
| Neither | 3 | 11 | 1 | 9 | 24 |
| % subject/spouse agreement | 71% | 94% | 67% | 88% | 85% |
| Spouse a smoker during year of hospital interview according to: | | | | | |
| Subject and spouse | 1 | 6 | 2 | 4 | 13 |
| Only subject | 0 | 0 | 0 | 1 | 1 |
| Only spouse | 1 | 0 | 0 | 0 | 1 |
| Neither | 5 | 12 | 7 | 20 | 44 |
| % subject/spouse agreement 86% | 86% | 100% | 100% | 96% | 97% |

spouses (3%) in respect of smoking during the year of hospital interview. There was no consistent pattern in the direction of discrepancy.

Table IV summarises the results of analyses carried out relating 7 indices of passive smoke exposure recorded in the hospital interviews to risk of lung cancer among lifelong non-smokers. Here the controls used for comparison are all never smoking patients with diseases classified as definitely or probably not associated with smoking who completed the passive smoking questionnaire.

Overall the results showed no evidence of an effect of passive smoking on lung cancer incidence among lifelong non-smokers. In male patients, relative risks were increased for some of the indices but numbers of cases were small and none of the differences approached statistical significance. In females, where numbers of cases were larger, such trends as existed tended to be negative and indeed were marginally significantly negative ($P<0.05$) for passive smoking during travel and during leisure. For the combined sexes no differences or trends were statistically significant at the 95% confidence level; such trends as existed tending to be slightly negative. The relative risk in relation to the spouse smoking during the whole of the marriage was estimated to be 0.80 for the sexes combined, with 95% confidence limits of 0.43 to 1.50. Standardisation for working in a dusty job, the variable apart from smoking found to have the strongest association with lung cancer risk in the analyses described in Alderson *et al.* (1985), did not affect the conclusion that passive smoking was not associated with risk of lung cancer among never smokers in our study.

### *Chronic bronchitis, ischaemic heart disease and stroke*

Analyses similar to that shown in Table IV for lung cancer were also carried out for chronic bronchitis, ischaemic heart disease and stroke. Illustrative results for two of the indices are presented in Table V.

No significant relationship of any index of passive smoking to risk of the 3 diseases was seen. For the sexes combined, the relative risk in relation to the spouse smoking during the whole of the marriage was 0.83 for chronic bronchitis (95% confidence limits 0.31–2.20), 1.03 for ischaemic heart disease (limits 0.65–1.62) and 0.90 for stroke (limits 0.53–1.52). For stroke there was, in both sexes, an approximate 2-fold increase in risk for patients with a combined passive smoke index that was high (score of 5 to 12) compared with those where it was low (score of 0 or 1). However, numbers of cases with a high score were low (14 males and 7 females) and even for the sexes combined, the relative risk estimate of 2.18 was not statistically significant (limits 0.86–5.48). In interpreting this finding, it should be noted that active smoking was not found to be clearly related to stroke in the main study (Alderson *et al.*, 1985), rendering a two-fold increase in relation to passive smoking *a priori* unlikely.

**Table IV** Relationship between various indices of passive smoke exposure and risk of lung cancer among lifelong non-smokers (standardised for age and, for spouse smoking, whether the marriage was ongoing or ended)

| *Passive smoke exposure index/level* | *Male patients* | | | *Female patients* | | | *Sexes combined* | | |
|---|---|---|---|---|---|---|---|---|---|
| | *Cases* | *Controls* | *R* | *Cases* | *Controls* | *R* | *Cases* | *Controls* | *R* |
| At home | | | | | | | | | |
| Not at all | 9 | 101 | 1 | 21 | 192 | 1 | 30 | 293 | 1 |
| Little | 2 | 21 | 1.22 | 6 | 65 | 0.92 | 8 | 86 | 0.98 |
| Average/a lot | 1 | 11 | 1.11 | 5 | 61 | 0.81 | 6 | 72 | 0.86 |
| At work | | | | | | | | | |
| Not at all | 3 | 40 | 1 | 12 | 113 | 1 | 15 | 153 | 1 |
| Little | 6 | 29 | 3.24 | 3 | 26 | 1.18 | 9 | 55 | 1.82 |
| Average/a lot | 1 | 29 | 0.46 | 0 | 19 | 0.0 | 1 | 48 | 0.19 |
| During travel | | | | | | | | | |
| Not at all | 8 | 101 | 1 | 28 | 238 | 1 | 36 | 339 | 1 |
| Little | 3 | 16 | 2.06 | 2 | 51 | 0.33 | 5 | 67 | 0.64 |
| Average/a lot | 0 | 13 | 0.00 | 0 | 13 | 0.00 Trend (negative) $P<0.05$ | 0 | 26 | 0.00 |
| During leisure | | | | | | | | | |
| Not at all | 3 | 45 | 1 | 15 | 116 | 1 | 18 | 161 | 1 |
| Little | 4 | 48 | 1.12 | 14 | 107 | 1.05 | 18 | 155 | 1.06 |
| Average/a lot | 5 | 39 | 3.18 | 2 | 95 | 0.18 Trend (negative) $P<0.05$ | 7 | 134 | 0.59 |
| Combined index[a] | | | | | | | | | |
| Score 0–1 | 1 | 27 | 1 | 10 | 75 | 1 | 11 | 102 | 1 |
| Score 2–4 | 7 | 55 | 4.34 | 5 | 61 | 0.63 | 12 | 116 | 1.08 |
| Score 5–12 | 2 | 15 | 3.20 | 0 | 21 | 0.00 | 2 | 36 | 0.50 |
| Spouse smoked man. cigs. in last 12 months | | | | | | | | | |
| No | 10 | 105 | 1 | 20 | 193 | 1 | 30 | 298 | 1 |
| Yes | 2 | 29 | 0.96 | 11 | 122 | 0.76 | 13 | 151 | 0.79 |
| Spouse smoked man. cigs. in whole of marriage | | | | | | | | | |
| No | 7 | 93 | 1 | 13 | 89 | 1 | 20 | 182 | 1 |
| Yes | 5 | 40 | 2.47 | 19 | 229 | 0.55 | 24 | 269 | 0.80 |

[a]Based on sum of 0 = not at all, 1 = little, 2 = average, 3 = a lot for at home, at work, during travel, during leisure.

### Discussion

Over the past 4 years there has been considerable research interest in the relationship between passive smoking and risk of lung cancer in nonsmokers. While some studies have claimed a positive effect (Hirayama, 1981; Trichopoulos *et al.*, 1981; Correa *et al.*, 1983; Garfinkel *et al.*, 1985; Gillis *et al.*, 1984; Knoth *et al.*, 1983), others (Buffler *et al.*, 1984; Chan, 1982; Garfinkel, 1981; Kabat and Wynder, 1984; Koo *et al.*, 1984) have found no significant relationship. Relative risks of lung cancer for non-smoking women married to smokers compared to non-smoking women married to non-smokers range from somewhat over 2 in the Trichopoulos and Correa studies to around 0.75 in the Buffler and Chan studies. The weighted relative risk from these studies has been estimated by us as approximately 1.3. While there is, therefore, a tendency for a small positive *association* between passive smoking and lung cancer, recent reviews of these data (Lee, 1984; Lehnert *et al.*, 1984) have concluded that overall there is no reliable scientific evidence of a causal relationship between passive smoking and lung cancer. In these reviews a number of general points have been made.

First, dosimetric studies have shown that, in cigarette-equivalent terms, passive smoking only results in a relatively small exposure to the non-smoker. Hugod *et al.* (1978), for example, showed that even under quite extreme conditions the time taken for a non-smoker to inhale the equivalent of

**Table V** Relationship between two indices of passive smoke exposure and risk of chronic bronchitis, ischaemic heart disease and stroke among lifelong non-smokers (standardised for age and, for spouse smoking, whether the marriage was ongoing or ended)

| *Passive smoke exposure index/level* | *Male patients* | | | *Female patients* | | | *Sexes combined* | | |
|---|---|---|---|---|---|---|---|---|---|
| | *Cases* | *Controls* | *R* | *Cases* | *Controls* | *R* | *Cases* | *Controls* | *R* |
| *Chronic bronchitis* | | | | | | | | | |
| Combined index[a] | | | | | | | | | |
| Score 0–1 | 1 | 27 | 1 | 7 | 75 | 1 | 8 | 102 | 1 |
| Score 2–4 | 2 | 55 | 0.83 | 4 | 61 | 1.05 | 6 | 116 | 1.00 |
| Score 5–12 | 1 | 15 | 1.90 | 1 | 21 | 1.03 | 2 | 36 | 1.30 |
| Spouse smoked man. cigs. in whole of marriage | | | | | | | | | |
| No | 8 | 93 | 1 | 4 | 89 | 1 | 12 | 182 | 1 |
| Yes | 1 | 40 | 0.34 | 13 | 229 | 1.22 | 14 | 269 | 0.83 |
| *Ischaemic heart disease* | | | | | | | | | |
| Combined index[a] | | | | | | | | | |
| Score 0–1 | 15 | 27 | 1 | 23 | 75 | 1 | 38 | 102 | 1 |
| Score 2–4 | 12 | 55 | 0.43 | 9 | 61 | 0.59 | 21 | 116 | 0.52 |
| Score 5–12 | 3 | 15 | 0.43 | 4 | 21 | 0.81 | 7 | 36 | 0.61 |
| Spouse smoked man. cigs. in whole of marriage | | | | | | | | | |
| No | 26 | 93 | 1 | 22 | 89 | 1 | 48 | 182 | 1 |
| Yes | 15 | 40 | 1.24 | 55 | 229 | 0.93 | 70 | 269 | 1.03 |
| *Stroke* | | | | | | | | | |
| Combined index[a] | | | | | | | | | |
| Score 0–1 | 5 | 27 | 1 | 19 | 75 | 1 | 24 | 102 | 1 |
| Score 2–4 | 10 | 55 | 1.24 | 10 | 61 | 0.86 | 20 | 116 | 0.97 |
| Score 5–12 | 4 | 15 | 1.77 | 7 | 21 | 2.44 | 11 | 36 | 2.18 |
| Spouse smoked man. cigs. in whole of marriage | | | | | | | | | |
| No | 18 | 93 | 1 | 19 | 89 | 1 | 37 | 182 | 1 |
| Yes | 6 | 40 | 0.84 | 49 | 229 | 0.92 | 55 | 269 | 0.90 |

[a]Based on sum of 0 = not at all, 1 = little, 2 = average, 3 = a lot for at home, at work, during travel, during leisure.

one cigarette would be 11 hours as regards particulate matter and 50 hours as regards nicotine. Similarly, Jarvis *et al.* (1985) have shown that the increase in salivary cotinine in relation to passive smoke exposure is less than 1% of that in relation to active smoke exposure. Extrapolating linearly from the 10-fold relative risk of lung cancer in relation to active smoking would therefore predict a relative risk in relation to passive smoking less than 1.1, while a quadratic extrapolation, as suggested by Doll and Peto (1978) would predict a lower risk still. The conflict between the dose and the claimed response is particularly clear for the results of Hirayama (1981) who found a similar effect on lung cancer for passive smoking as for active smoking of 5 cigarettes a day.

Second, all the studies suffer from weak exposure data, most studies only obtaining information on the spouse's smoking habits and none obtaining objective data by measurement of ambient levels of smoke constituents in the air of the home or workplace and/or of concentrations of constituents in body fluids.

Third, no studies adequately take into account the possibility that misclassification of active smokers as non-smokers may have consistently biased relative risk estimates upward. Active smokers have a high relative risk of lung cancer and spouses' smoking habits are positively correlated. Because of this, it can be shown that if a relatively small proportion of smokers deny smoking, this results in an *apparent* elevation in risk of lung cancer in 'non-smokers' married to smokers compared to 'non-smokers' married to non-smokers, even when no *true* effect of passive smoking exists. A demonstration that this source of bias is of real importance can be found in the study of Garfinkel *et al.* (1985). Based on unvalidated smoking data taken from hospital notes, a relative risk of lung cancer in relation to husband's smoking at home of 1.66 was calculated, with relative risks of at least 1.3 seen in relation to each level of husband's cigarette smoking and in relation to husband's cigar and pipe smoking. When additional sources of information on smoking habits were used, the overall relative risk was reduced to a marginally significant 1.31 with an elevated risk only really discernible in relation to heavy cigarette smoking by the husband. Even here, it is notable that the elevation in risk was not evident when smoking data were obtained from the subject or her spouse directly, but was only evident when the data were obtained from the daughter or son or another informant, i.e. from those people who were less likely to have known the full smoking history. The lower relative risk may still have arisen wholly or partly as a bias resulting from misclassification of smoking habits.

Fourth, many of the studies are open to specific criticisms. For example, the conclusion of Gillis *et al.* (1984) that male lung cancer deaths in non-smokers rose from 4 per 10,000 in those not exposed to passive smoke to 13 per 10,000 in those who were exposed was based on a total of only 6(!) deaths and was not statistically significant. Also the claim by Knoth *et al.* (1983) of a relationship between passive smoking and lung cancer in non-smoking women was based simply on the observation that the proportion of female non-smoking lung cancer patients living together with a smoker exceeded the proportion of male smokers as reported in the previous microcensus, ignoring *inter alia* the fact that in many families women live with more than just their husbands.

In the present study no significant relationship of passive smoking to lung cancer incidence in lifelong non-smokers was seen, either in the analyses based on the information collected in hospital or in subsequent inquiry of the spouses or both. It must be pointed out, however, that the number of lung cancer patients who had never smoked was rather small so that, though our findings are consistent with passive smoking having no effect on lung cancer risk at all, they do not exclude the possibility of a small increase in risk, though the upper 95% confidence limit of 1.50 for the estimate of 0.80 (Table IV) in relation to the spouse smoking during the whole of the marriage is not consistent with some of the larger increases claimed by Hirayama (1981, 1984) Trichopoulos *et al.* (1981, 1983) and Correa *et al.* (1983).

Though the number of lung cancer patients who had never smoked is small, varying around 30–50 depending on the analysis, this number is not very different from that reported in a number of other studies, e.g. the findings of Correa *et al.* (1983) were based on only 30, while those of Trichopoulos *et al.* (1981), even when updated (Trichopoulos *et al.*, 1983) were based on only 77. The difficulty of obtaining an adequate sample size is underlined when one considers that in our study the 44 never smoking lung cancer patients who completed passive smoking questionnaires in hospital were extracted from a total of 792 lung cancer patients. It would need a very large research effort to increase precision substantially, and even then one would have to take care that the magnitude of any biases did not exceed the magnitude of the effect one was looking for.

The two major prospective studies which have so far reported findings on passive smoking (Hirayama, 1981; Garfinkel, 1981) were not actually designed to investigate this issue and, as a result, could only use spouse's smoking as an index of exposure. Our study, on the other hand, though not able to monitor exposure objectively, as would have been preferable, was able to look at passive smoking in a wider context, by asking about the extent of exposure at home, at work, during travel and at leisure. Although the answers to these questions were subjective, and could have exhibited some bias, their inclusion perhaps allows greater confidence in the conclusions.

It was interesting that, of the 59 patients for whom spouse's cigarette smoking habits were obtained from both the spouse and the patients, there were 9 (15%) patients for whom there was disagreement as to whether the spouse had been a smoker at some time during the marriage. It seems reasonable to suppose that some of these were in fact smokers and may have been erroneously classified as non-smokers had only one source of information been used. It was also noteworthy that there was quite a strong correlation in our study between active and passive smoking. As illustrated in Table VI, current smokers were considerably more likely to be exposed to passive smoke exposure at home (from sources other than their own cigarettes) than were never or ex-smokers. As noted above, this correlation, coupled with some misclassification of smokers as non-smokers, may spuriously inflate the estimate of risk related to passive smoking. It is important to carry out further studies to obtain more accurate information on reliability of statements about smoking habits because of this possibility of bias.

Little other evidence is available concerning the relationship between passive smoking and risk of the other smoking-associated diseases in (adult) non-smokers and much of this is open to criticism. In his original paper, Hirayama (1981) presented relative risks of death for various diseases for non-smoking women according to the husband's smoking habits. Based on a total of 66 deaths, a slight positive trend for emphysema and asthma was not significant, while, based on a total of 406 deaths, no indication of a trend at all was seen for ischaemic heart disease. In a later paper, based on

**Table VI** Relative odds of having passive smoke exposure at home according to patient's own manufactured cigarette smoking habits (standardised for age: base – combined class 1 and 2 controls)

| | *Relative odds (95% confidence limits)* | |
|---|---|---|
| *Own smoking habits* | *Male* | *Female* |
| Never | 1 | 1 |
| Ex | 1.25(0.86–1.81) | 1.26(0.86–1.85) |
| Current | 4.00(2.67–5.98) | 2.51(1.74–3.62) |
| Chi-squared for trend (2 df) | 57.81 | 25.34 |
| *P* | <0.001 | <0.001 |

only a further 88 ischaemic heart disease deaths, Hirayama (1984) reported a slight positive trend in risk, but this was not statistically significant. Garland *et al.* (1985), in a small prospective study, reported a 15-fold higher risk of ischaemic heart disease in non-smoking Californian women whose husbands were current or former smokers compared with those whose husbands were never smokers, but this enormous and implausible relative risk was only significant at the 90% confidence level and had very wide confidence limits, being based on only 2 deaths in women whose husbands were current smokers. Sandler *et al.* (1985), in a case-control study carried out in North Carolina, reported a strong relationship between risk of cancer of all sites and passive smoking. This study has been criticised by Lee (1985) who notes that it is basically implausible that passive smoking should increase risk of cancers not associated with active smoking. Lee also criticised the method of analysis, showing that no association with cancer risk would be found if a more standard method of analysis was used. Vanderbroucke *et al.* (1984), based on a 25 year follow-up of 1,070 Amsterdam married couples, recently reported that passive smoking was associated with some decrease in total mortality.

There is evidence indicating that young children whose parents smoke have an excess incidence of respiratory symptoms and some reduction in pulmonary function. Reviewing this evidence, Lee (1984) noted that the interpretation of the association is fraught with difficulties and that other possible explanations, including social class related factors, parental negelct, nutrition, cross-infection and smoking during pregnancy, had not been taken into account adequately, so that a causal effect of passive smoking could not be inferred. The relevance of these findings to chronic bronchitis or other diseases in adults is in any case not clear.

Our analyses showed no significant effect of passive smoking on lifelong non-smokers as regards risk of chronic bronchitis, ischaemic heart disease or stroke. In all the analyses relating the various indices of passive smoke exposure to these diseases, no significant differences were seen and slight decreases in risk were as common as slight increases.

While more data would be desirable for these diseases, lung cancer continues to be the major smoking associated disease for which passive smoking comes under suspicion. Since all the difficulties of carrying out good research have clearly still not yet been overcome, further research is certainly needed. Our findings appear consistent with the general view, based on all the available evidence, that any effect of passive smoking on risk of lung cancer or other smoking-associated diseases is at most quite small, if it exists at all. The marked increases in risk noted in some studies are more likely to be a result of bias in the study design than of a true effect of passive smoking.

Any views expressed in this paper are those of the authors and not of any other person or company.

This study was funded by the Tobacco Research Council (now Tobacco Advisory Council), to whom we are most grateful. Dr Alderson was the holder of the Cancer Research Campaign endowed Chair of Epidemiology at the Institute of Cancer Research during the period of the study design and field work.

Mr. I. Marks from Research Surveys of Great Britain provided advice in the planning phase and was responsible for the interviewers' vital contribution to the study. We thank the many clinicians at the 46 participating hospitals who permitted us to contact their patients and all the patients and spouses who answered the questions.

Dr R. Wang, who held a British Council award for the period 1980–1983, as well as a number of other colleagues provided useful advice at various stages of the study.

Mrs B.A. Forey provided invaluable assistance in carrying out the statistical analyses.

**References**

ALDERSON, M.R., LEE, P.N. & WANG, R. (1985). Risks of lung cancer, chronic bronchitis, ischaemic heart disease and stroke in relation to type of cigarette smoked. *J. Epidem. Comm. Hlth.*, **39**, 286.

BRESLOW, N.E. & DAY, N.E. (1980). *Statistical Methods in Cancer Research Vol 1 – The Analysis of Case-control Studies*. International Agency for Research on Cancer; Lyon.

BUFFLER, P.A., PICKLE, L.W., MASON, T.J. & CONTANT, C. (1984). The causes of lung cancer in Texas. In *Lung Cancer. Causes and Prevention*, Mizell, M. & Correa, P. (eds). Verlag Chemie International Inc.

CHAN, W.C. (1982). Zahlen aus Hongkong. *Munch. Med. Woch.*, **124**, 16.

CORREA, P., PICKLE, L.W., FONTHAM, E., LIN, Y. & HAENSZEL, W. (1983). Passive smoking and lung cancer. *Lancet*, **ii**, 595.

DOLL, R. & PETO, R. (1978). Cigarette smoking and bronchial carcinoma: dose and time relationships among regular smokers and lifelong non-smokers. *J. Epidem. Comm. Hlth*, **32**, 303.

GARFINKEL, L. (1981). Time trends in lung cancer mortality among non-smokers and a note on passive smoking. *J. Natl Cancer. Inst.*, **66**, 1061.

GARFINKEL, L., AUERBACH, O. & JOUBERT, L. (1985). Involuntary smoking and lung cancer: A case-control study. *J. Natl Cancer Inst.*, **75**, 463.

GARLAND, C., BARRETT-CONNOR, E., SUAREZ, L., CRIQUI, M.H. & WINGARD, D.L. (1985). Effects of passive smoking on ischemic heart disease mortality of non-smokers. A prospective study. *Amer. J. Epidem.*, **121**, 645.

GILLIS, C.R., HOLE, D.J., HAWTHORNE, V.M. & BOYLE, P. (1984). The effect of environmental tobacco smoke in two urban communities in the west of Scotland. *Europ. J. Resp. Dis.*, **65**, (Suppl. 133), 121.

HIRAYAMA, T. (1981). Non-smoking wives of heavy smokers have a higher risk of lung cancer: a study from Japan. *Br. Med. J.*, **282**, 183.

HIRAYAMA, T. (1984). Lung cancer in Japan: effects of nutrition and passive smoking. In *Lung Cancer, Causes and Prevention*. Mizell, M. & Correa, P. (eds). Verlag Chemie International Inc.

HUGOD, C., HAWKINS, L.H. & ASTRUP, P. (1978). Exposure of passive smokers to tobacco smoke constituents. *Int. Arch. Occup. Environ. Hlth*, **42**, 21.

JARVIS, M.J., RUSSELL, M.A.H., FEYERABEND, C. & 4 others (1985). Passive exposure to tobacco smoke: saliva cotinine concentrations in a representative population sample of non-smoking school-children. *Br. Med. J.*, **291**, 927.

KABAT, G.C. & WYNDER, E.L. (1984). Lung cancer in non-smokers. *Cancer*, **53**, 1214.

KNOTH, A., BOHN, H. & SCHMIDT, F. (1983). Passive smoking as cause of lung cancer in female non-smokers. *Med. Klin.*, **78**, 54.

KOO, L.C., HO, JH-C. & SAW, D. (1984). Is passive smoking an added risk factor for lung cancer in Chinese women? *J. Exp. Clin. Cancer Res.*, **3**, 277.

LEE, P.N. (1984). Passive Smoking. In *Smoking and the Lung*. Cumming, G & Bonsignore, G. (eds). Plenum Publishing Corporation.

LEE, P.N. (1985). Lifetime passive smoking and cancer risk. *Lancet*, **i**, 144.

LEHNERT, G., GARFINKEL, L., HIRAYAMA, T. & 4 others. (1984). Round table discussion. *Prev. Med.*, **13**, 730.

SANDLER, D.P., WILCOX, A.J. & EVERSON, R.B. (1985). Cumulative effects of lifetime smoking on cancer risk. *Lancet*, **i**, 312.

TRICHOPOULOS, D., KALANDIDI, A., SPARROS, L. & MACMAHON, B. (1981). Lung cancer and passive smoking. *Int. J. Cancer*, **27**, 1.

TRICHOPOULOS, D., KALANDIDI, A. & SPARROS, L. (1983). Lung cancer and passive smoking: Conclusion of Greek study. *Lancet*, **ii**, 677.

VANDERBROUCKE, J.P., VERHEESEN, J.H.H., DE BRUIN, A., MAURITZ, B.J. VAN DER HEIDE-WESSEL, C. & VAN DER HEIDE, R.M. (1984). Active and passive smoking in married couples: results of 25 year follow up. *Br. Med. J.*, **288**, 1081.